# Sensitivity of the Photosynthetic Apparatus in Maize and Sorghum under Different Drought Levels

**DOI:** 10.3390/plants12091863

**Published:** 2023-04-30

**Authors:** Martin Stefanov, Georgi Rashkov, Preslava Borisova, Emilia Apostolova

**Affiliations:** Institute of Biophysics and Biomedical Engineering, Bulgarian Academy of Sciences, 1113 Sofia, Bulgaria; martin_12.1989@abv.bg (M.S.); megajorko@abv.bg (G.R.); preslavab12345@gmail.com (P.B.)

**Keywords:** chlorophyll fluorescence, PEG treatment, P_700_ photooxidation, pigment composition, membrane injury, maize, sorghum, relative water content

## Abstract

Drought is one of the main environmental stress factors affecting plant growth and yield. The impact of different PEG concentrations on the photosynthetic performance of maize (*Zea mays* L. Mayflower) and sorghum (*Sorghum bicolor* L. Foehn) was investigated. The activity of the photosynthetic apparatus was assessed using chlorophyll fluorescence (PAM and JIP test) and photooxidation of P_700_. The data revealed that water deficiency decreased the photochemical quenching (qP), the ratio of photochemical to nonphotochemical processes (Fv/Fo), the effective quantum yield of the photochemical energy conversion in PSII (Φ_PSII_), the rate of the electron transport (ETR), and the performance indexes PI_total_ and PI_ABS_, as the impact was stronger in sorghum than in maize and depended on drought level. The PSI photochemistry (P_700_ photooxidation) in sorghum was inhibited after the application of all studied drought levels, while in maize, it was registered only after treatment with higher PEG concentrations (30% and 40%). Enhanced regulated energy losses (Φ_NPQ_) and activation of the state transition under drought were also observed in maize, while in sorghum, an increase mainly in nonregulated energy losses (Φ_NO_). A decrease in pigment content and relative water content and an increase in membrane damage were also registered after PEG treatment. The experimental results showed better drought tolerance of maize than sorghum. This study provides new information about the role of regulated energy losses and state transition for the protection of the photosynthetic apparatus under drought and might be a practical approach to the determination of the drought tolerance of plants.

## 1. Introduction

Plants are subjected to the action of various environmental stress factors during their development [1]. Among the environmental stresses, drought is attracting increasing attention due to its strong negative effect on plant biomass and a significant decrease in crop yield [2,3]. This stress is a natural climatic factor affecting plant growth and development, and it occurs in almost all temperate zones, as its effects depend on the frequency, severity, and duration [2,4]. The drought will become more frequent and last longer as a result of upcoming climate changes, making this one of the most serious concerns of the twenty-first century [5].

This environmental stress has a significant impact on all essential plant processes, including photosynthesis, respiration, and mineral nutrient intake, limiting the supply of photosynthetic assimilates and energy to the plant [5,6]. Drought stress influences the morphological and anatomical characteristics and the photosynthetic rate of drought-sensitive plants [7]. As a result of climate change, global warming, and an increase in ultraviolet (UV) radiation, especially the UV-B, the negative impact on plant photosynthesis has intensified [1,8]. The combination of solar UV and water deficit influences leaf morphology and has species-specific effects [9]. It has been also shown that exposure of soybean and maize to UV-B under drought leads to an increased membrane damage and a reduction of the chlorophyll content, as well as an inhibition of the photosynthetic rate, in comparison with the effect of drought alone [10]. On the other hand, the combined effect of drought and heat is higher than when taken individually [8].

Many plants have improved their resistance mechanisms to decrease the negative effects of drought stress, but these mechanisms are different and depend on the plant species [11]. First, drought inhibits gas exchange, impairs the stomatal function, and causes the overproduction of the reactive oxygen species (ROS), which lead to oxidative stress [12]. Second, a decrease in water content affects the cell division, leaf surface expansion, stem growth, and root cell proliferation [13]. All of these changes significantly impair plant development and may result in the death of plants after prolonged drought exposure [5,14]. The overproduction of ROS in plants causes damage to proteins, lipids, carbohydrates, and nucleic acids [14,15]. Plants are strongly influenced by oxidative damage, which causes changes in chloroplasts and in the structure and functions of thylakoid membranes [14,16].

The influence of drought stress on the morphological, biochemical, and physiological processes in plants [17] strongly affects photosynthetic performance, which is very important for plant growth and productivity under drought [18]. It has been shown that there is an alteration in protein–protein interactions, increase in protein aggregation, and denaturation [19]. These changes correspond to an inhibition of the electron transport activity of the photosynthetic apparatus and a decrease in net photosynthesis [18,20,21,22]. Furthermore, the low CO_2_ uptake caused by stomatal closure is the primary stomatal dependent factor that reduces the photosynthetic rate due to the decreased activity of CO_2_ reduction enzymes (Calvin cycle). The downregulation of the dark reactions may result in photosynthetic imbalances between light and dark processes, which causes an over-reduction of the photosynthetic electron transport chain [23,24,25,26].

Another factor influencing the inhibition of the photosynthetic rate is a reduction of the chlorophyll content (Chl) under water deficit [27], which affects the light harvest ability [27]. Furthermore, the changes in pigment composition vary depending on the drought tolerance of the plants [15]. Previous investigations revealed that the reduction of Chl *b* is bigger than that of Chl *a* [28]. Additionally, some studies found an increase in chlorophyll content in *Vitis* hybrids and in *Avena sativa* after prolonged growth in water scarcity [29,30]. At the same time, drought has a smaller influence on carotenoid content than chlorophylls. It has also shown an increase in xanthophyll pigments, such as zeaxanthin and antheraxanthin. Upon exposure to drought, the functions of photosystem I (PSI) and photosystem II (PSII) and the electron transport from PSII to PSI are influenced depending on the drought tolerance of plant species [11]. A number of in vivo investigations have revealed that drought stress causes significant damage to the oxygen-evolving complex (OEC) [31,32], dissociation of the light-harvesting complex of PSII (LHCII) from the reaction centers of PSII [33], and D1 polypeptide degradation, which results in the donor and acceptor side changes of PSII and a decrease in its photochemical efficiency [28,34,35,36,37,38]. The levels of PSII reaction center proteins and the light-harvesting complexes of PSI (LHCI) and PSII (LHCII) diminished significantly owing to the water deficiency as a result of the influence on their biosynthesis and degradation [28,39]. It has also been shown that the PSII photochemical activity is more vulnerable to osmotic stress than the PSI activity [37,40]. Plants evolve different physiological, morphological, and biochemical adaptive traits to cope with the negative impact of drought [11]. Plants protect the photosynthetic apparatus by dissipating excess energy via nonphotochemical quenching, as xanthophyll-dependent energy dissipation is its main constituent in higher plants [41]. The stimulation of cyclic electron transport around PSI is another protective mechanism for the photosynthetic apparatus [37]. The activation of antioxidant enzymes (superoxide dismutase (SOD), catalase (CAT), ascorbate peroxidase (APX), etc.), which detoxify ROS molecules, and the synthesis of protective components (carotenoids, proline, flavonoids, anthocyanins, etc.) are also very important for the survival of plants under drought [42,43,44,45]. While the role of most nonenzymatic antioxidants is well studied, the role of anthocyanins under stress conditions is not fully understood. They are natural components that accumulate in plants under stress, and it is suggested that their main role is to mediate responses to stress [46].

Maize (*Zea mays* L.) and sorghum (*Sorghum bicolor* L.) are universal crops, widely grown throughout the world. Maize is the third most significant cereal crop. It is used for food and fodder, and its yield is strongly affected by drought [47]. Considering its importance, there is an increasing focus on the selection of maize hybrids that are resistant to drought [48]. Sorghum is a crop that is among the top 5 crops in the world and is the second most widespread crop in Africa. It is used for food and animal feed and also for industrial purposes [49], which makes sorghum an attractive crop [50]. When compared with other cereal crops, it is thought to be more resistant to a variety of conditions, such as heat, drought, salinity, and flooding [6]. Drought stress is considered to be the most frequent abiotic stress on sorghum in its principal production areas. Although sorghum and maize share some common physiological and morphological characteristics, they have different tolerances to abiotic stress [51,52,53,54,55]. Therefore, considerable attention has been given to understanding the effects of drought stress in sorghum and in maize, and their stress tolerance mechanisms, as part of efforts to develop tolerant cultivars and apply efficient strategies to alleviate stress [56].

It is crucial to evaluate the tolerance of significant agricultural crops and their capacity to adapt to dynamic environmental conditions, one of which is drought. On the other hand, research on the effects of drought on different plant species can contribute to the progress in breeding research of plant tolerant lines. It is well known that photosynthesis is very sensitive to abiotic stress factors [57]. In our previous study, we revealed that the parameters of primary photochemistry are sensitive to salt stress, and their changes strongly depend on plant salt tolerance [51,58]. We hypothesize that the drought-induced changes in the primary photochemistry of photosynthesis and the mechanisms involved in photosynthetic apparatus protection, which activate under drought, can be used to assess the drought tolerance of the plants. Moreover, the extent of drought-induced changes will determine the extent of their recovery in the postdrought period. For this purpose, we study the impact of PEG-induced drought in two plant species (maize and sorghum) with different drought sensitivities. This investigation examines the functions of the photosynthetic apparatus and the mechanisms of photosynthetic apparatus protection in maize (*Zea mays* L. Mayflower) and in sorghum (*Sorghum bicolor* L. Foehn) after treatment with different PEG concentrations (20%, 25%, 30%, and 40%) and the possibility of their recovery after the different levels of drought. The degree of recovery of plants after drought will give information to what level of drought the plants can restore without serious changes occurring in them. In addition, the pigment content, the stress markers, and membrane damage are also studied. The experimental results provide additional new information on the important role (relationship) of the mechanisms of photosynthetic apparatus protection and drought sensitivity of these crops.

## 2. Results

### 2.1. Pigment Composition

The influence of PEG-induced drought on the pigment composition in maize and sorghum and their recovery is shown in Table 1. Data revealed that the amounts of total chlorophylls were higher in sorghum than in maize in untreated (control) plants. PEG-induced drought led to a decrease in pigment content (Chl and Car), as significant changes were registered in plants treated with concentrations higher than 25% PEG. Moreover, the presence of 40% PEG in nutrient solution was lethal for sorghum plants. Experimental results showed that the treatment with 25% PEG and higher concentrations led to a smaller decrease in Car content compared with Chl in both species studied, but after the application of 20% PEG in the nutrient solution, the Car content was similar to the control plants. The treatment with 30% PEG led to the reduction in Chl amount by 52% in maize and 66% in sorghum, while the reduction in carotenoids was 40% and 37% in maize and in sorghum, respectively. The changes in pigment composition influence the Chl *a*/*b* ratio (Table 2). This ratio increases in all studied drought levels in sorghum and in maize. The Chl *a*/*b* ratio was higher after applying 30% PEG with 15% and 9% in maize and in sorghum, respectively. The drought-induced changes in the Car/Chl ratio were registered in sorghum after treatment with all studied PEG concentrations, while in maize, a negligible influence was only observed after applying 40% PEG (Table 2). After the recovery period, the pigment amount increased depending on the applied PEG concentrations (or drought level), and it was better after treatment with the smallest PEG concentration (20%) (Table 1). In addition, experimental results revealed that the ratios Chl *a*/*b* and Car/Chl were similar to the control variants with the exception of maize treated with 40% PEG.

### 2.2. Anthocyanins

**Figure 1:**
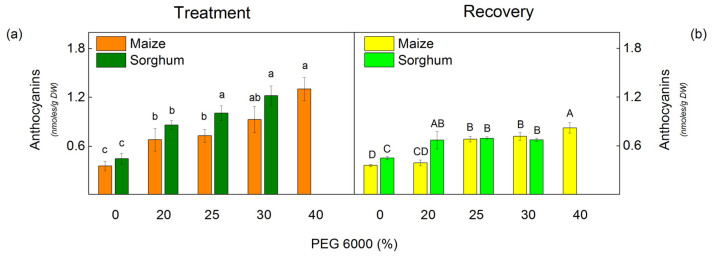
The amount of anthocyanins in maize (*Zea mays* L. Mayflower) and in sorghum (*Sorghum bicolor* L. Foehn) after PEG treatment (**a**) and after the recovery period (**b**) of the drought-treated plants. Mean values (±SE) were calculated from 8 independent measurements. Different letters indicate significant differences among treatments at *p* < 0.05 (lowercase for the plants after the treatment and uppercase for the plants after the recovery period).

The drought resulted in a strong accumulation of anthocyanins in both species studied (Figure 1). The level of anthocyanins increased with increasing PEG concentrations. The increase in these pigments was more pronounced in sorghum (by 125% for 25% PEG and by 172% for 30% PEG) than in maize (by 104% for 25% PEG and by 160% for 30% PEG) (Figure 1). After the recovery period, the amount of anthocyanins decreased in all studied variants, and in maize plants treated with 20% PEG, it was similar to the control.

### 2.3. Oxidative Stress Markers and Membrane Injury

**Figure 2:**
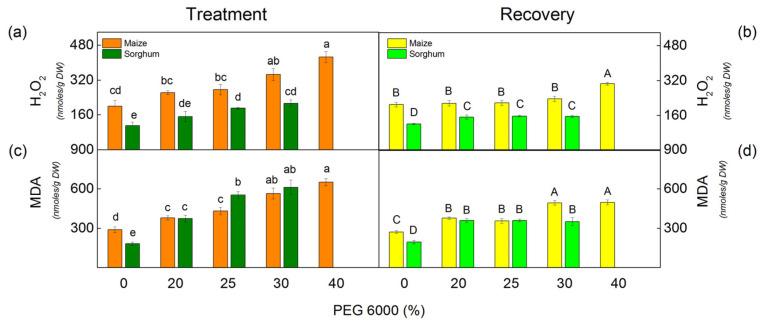
The amounts of H_2_O_2_ (**a**,**b**) and MDA (c,d) in maize (*Zea mays* L. Mayflower) and in sorghum (*Sorghum bicolor* L. Foehn) after PEG treatment (**a**,**c**) and after the recovery period (**b**,**d**) of the drought-treated plants. Mean values (±SE) were calculated from 8 independent measurements. Different letters indicate significant differences among treatments at *p* < 0.05 (lowercase for the plants after the treatment and uppercase for the plants after the recovery period).

The drought resulted in an increase of H_2_O_2_ in sorghum plants treated with all studied PEG concentrations, while in maize plants, only treated with 30% and 40% PEG (Figure 2). Moreover, the increase was more pronounced in sorghum than in maize. After treatment with 30% PEG, the rise was 92% in sorghum and 73% in maize (Figure 2). Data also revealed an increase in H_2_O_2_ content by 113% in maize after applying 40% PEG, while this concentration was lethal for sorghum (Figure 2a).

The level of lipid peroxidation (assessed by MDA content) corresponds to membrane damage. Drought induced an increase in MDA content in all studied variants in comparison with untreated plants, as the changes depend on the applied PEG concentrations (Figure 2). A strong increase was registered after treatment with 30% and 40% PEG in maize and 25% and 30% PEG in sorghum.

After the recovery period of the drought-stressed plants, MDA and H_2_O_2_ content decreased in both studied species, but their amounts were higher than the respective controls (Figure 2).

The membrane injury index (MII) characterized the membrane integrity, and it is a quick marker for determining drought tolerance [59]. The MII increased after PEG treatment in both studied species (Table 3). The increase in this parameter was more pronounced in sorghum than in maize; i.e., the drought-induced changes in membrane integrity were bigger in sorghum in comparison with maize. After the recovery period, the MII decreased in all studied variants (Table 3). In addition, data revealed that the MII values were smaller in maize than in sorghum for plants treated with PEG concentration from 20% to 30% (Table 3).

### 2.4. Relative Water Content

**Figure 3:**
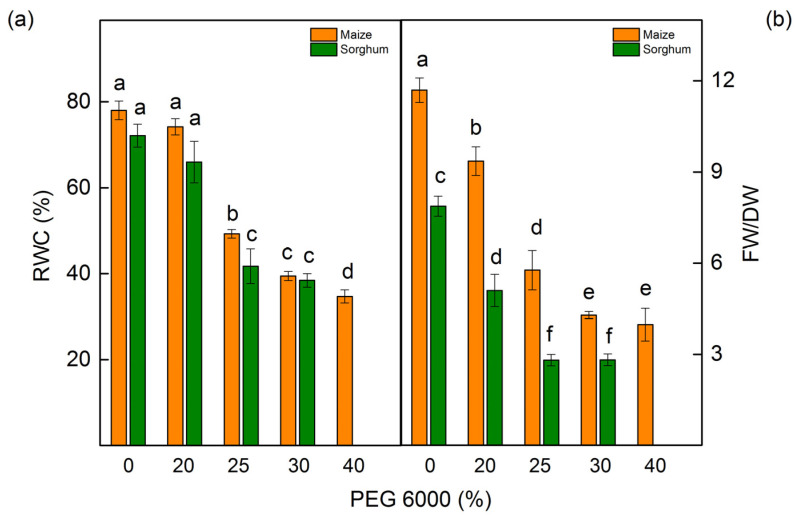
Relative water content (RWC) (**a**) and the ratio of fresh weight/dry weight (FW/DW) (**b**) in maize (*Zea mays* L. Mayflower) and in sorghum (*Sorghum bicolor* L. Foehn) treated with different PEG concentrations. Mean values (±SE) were calculated from 8 independent measurements. Significant differences between treatments at *p* < 0.05 are indicated by different letters for the respective parameter.

The measurements of the relative water content (RWC) revealed that drought influences this parameter depending on the applying PEG concentrations (Figure 3a). Some decrease in water content was registered after treatment with 25% PEG and higher concentrations in both studied species. The drought-induced changes led to a decrease in the FW/DW ratio (Figure 3b). The decrease of this ratio in both studied species was after treatment with all studied PEG concentrations (Figure 3b).

### 2.5. PAM Chlorophyll Fluorescence

**Figure 4:**
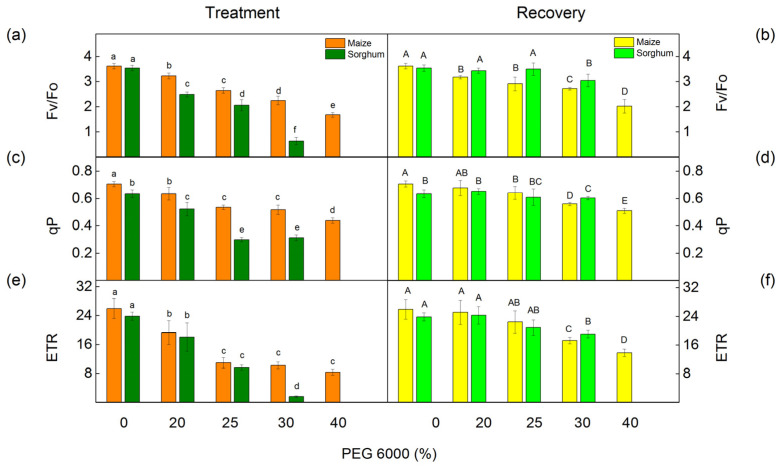
PAM chlorophyll fluorescence parameters in maize (*Zea mays* L. Mayflower) and in sorghum (*Sorghum bicolor* L. Foehn) after PEG treatment and after the recovery period. The ratio of photochemical to nonphotochemical processes, Fv/Fo (**a**,**b**); the photochemical quenching qP (**c**,**d**); and the rate of linear electron transport, ETR (**e**,**f**). Values (±SE) were calculated from 8 independent measurements. Different letters indicate significant differences for the respective parameters at *p* < 0.05 (lowercase for the plants after the treatment and uppercase for the plants after the recovery period).

The PAM chlorophyll fluorescence measurements showed that the PEG treatment influenced the ratio of photochemical to nonphotochemical processes in PSII (Fv/Fo), the photochemical quenching (qP), and the electron transport rate (ETR) (Figure 4). Small changes in these parameters were registered even after treatment with the lowest PEG concentration (20%). Data also showed that the impact of these parameters was bigger in sorghum than in maize (Figure 4a,c,e). After the period of the recovery, these parameters increased in comparison with the drought-treated plants (Figure 4b,d,f).

**Figure 5:**
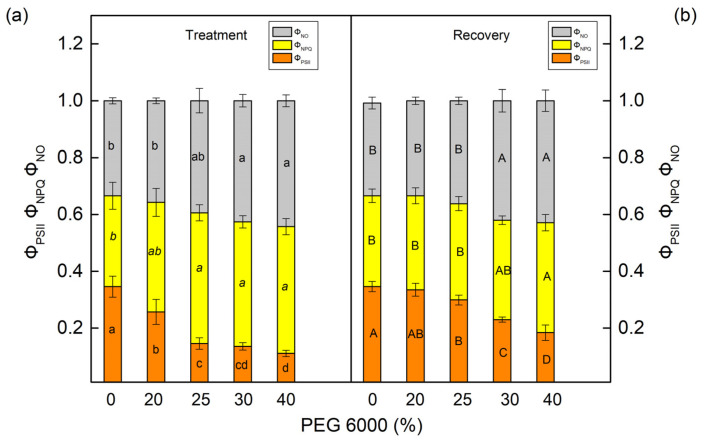
PAM chlorophyll fluorescence parameters in maize (*Zea mays* L. Mayflower) (**a,b**) after treatment with different PEG concentrations (**a**) and after period of recovery (**b**). The effective photochemical energy conversion quantum yield of PSII (Φ_PSII_). The ratios of nonregulated (Φ_NO_) and regulated (Φ_NPQ_) energy loss in PSII. Values (±SE) were calculated from 8 independent measurements. Different letters (lowercase for the plants after the treatment and uppercase for the plants after the recovery period) indicate significant differences for the respective parameters at *p* < 0.05.

**Figure 6:**
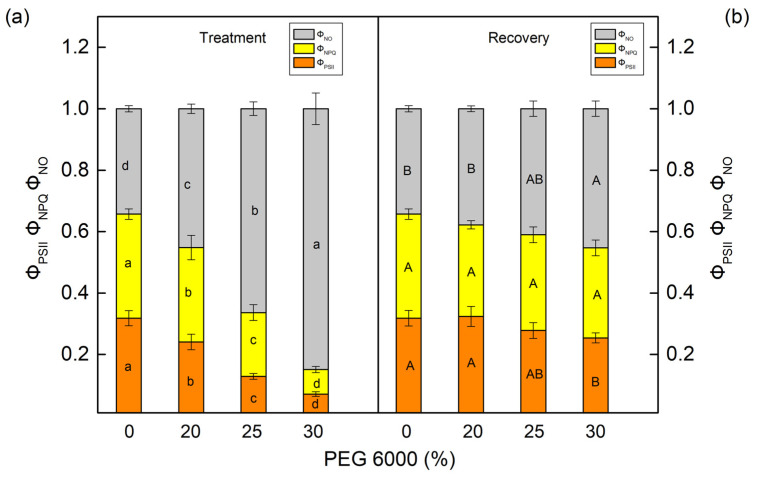
The parameters of the PAM chlorophyll fluorescence in sorghum (*Sorghum bicolor* L. Foehn) (**a**,**b**) after treatment with different PEG concentrations (**a**) and after period of recovery (**b**). The effective photochemical energy conversion quantum yield of PSII, Φ_PSII_. The ratios of nonregulated (Φ_NO_) and regulated (Φ_NPQ_) energy loss in PSII. Mean values (±SE) were calculated from 8 independent measurements. Different letters (lowercase for the plants after the treatment and uppercase for the plants after the recovery period) indicate significant differences for the respective parameters at *p* < 0.05.

The PEG treatment influenced the effective quantum yield of the photochemical energy conversion in PSII (Φ_PSII_) and the quantum yields of regulated (Φ_NPQ_) and nonregulated (Φ_NO_) energy losses in PSII (Figure 5 and Figure 6). These parameters were strongly influenced in both studied species after treatment with higher PEG concentrations (25% and higher). The parameter Φ_PSII_ decreased by 68% in maize and by 84% in sorghum after treatment with 30% PEG. At the same time, energy losses in PSII (the sum of Φ_NPQ_ and Φ_NO_) increased in both studied species. Data also showed that Φ_NPQ_ increased in maize, but in sorghum, this parameter decreased. The drought-induced changes in nonregulated energy losses (Φ_NO_) were smaller in maize, while in sorghum, these losses strongly increased (Figure 5a and Figure 6a). After the period of recovery, Φ_PSII_ increased and energy losses (Φ_NPQ_ and Φ_NO_) decreased in all studied variants (Figure 5b and Figure 6b).

**Figure 7:**
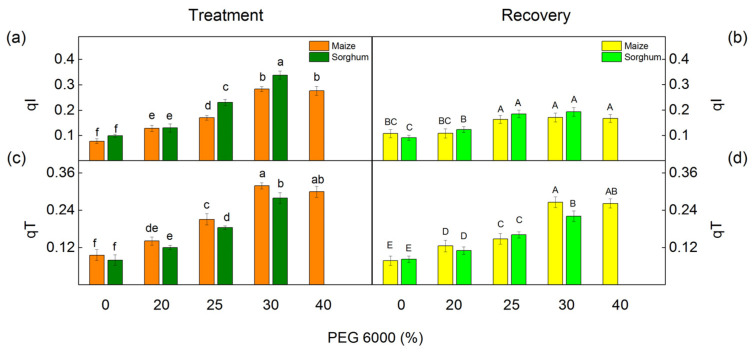
Components of the nonphotochemical quenching in maize (*Zea mays* L. Mayflower) and in sorghum (*Sorghum bicolor* L. Foehn) after PEG treatment (**a**,**c**) and after period of recovery (**b**,**d**). Photoinhibitory component, qI (**a**,**b**); state transition component, qT (**c**,**d**). Values (±SE) were calculated from 8 independent measurements. Different letters indicate significant differences for the respective parameters at *p* < 0.05 (lowercase for the plants after the treatment and uppercase for the plants after the recovery period).

More information on nonphotochemical quenching mechanisms is revealed by the following components: the state transition quenching (qT) caused by reversible phosphorylation of LHCII and the photoinhibition-induced quenching of the PSII reaction center (qI). The effects of different concentrations of PEG on these components are shown in Figure 7. Under drought stress, an increase in both investigated components was found in maize and in sorghum. Significant increases in component qI were established in sorghum and in qT in maize after PEG exposure as the effects were bigger after treatment with 30% PEG (Figure 7a,c). After the recovery period, these components (qI and qT) decreased, but the values remained bigger compared with the control values (Figure 7b,d).

### 2.6. Chlorophyll Fluorescence Induction

Chlorophyll fluorescence induction was also used to assess the impact of drought on photosynthetic performance. The selected JIP parameters (ETo/RC, REo/RC, φEo, φRo, N, PI_ABS_, PI_total_), which give additional information for the drought-induced effects in the primary photosynthetic reactions, were calculated.

**Figure 8:**
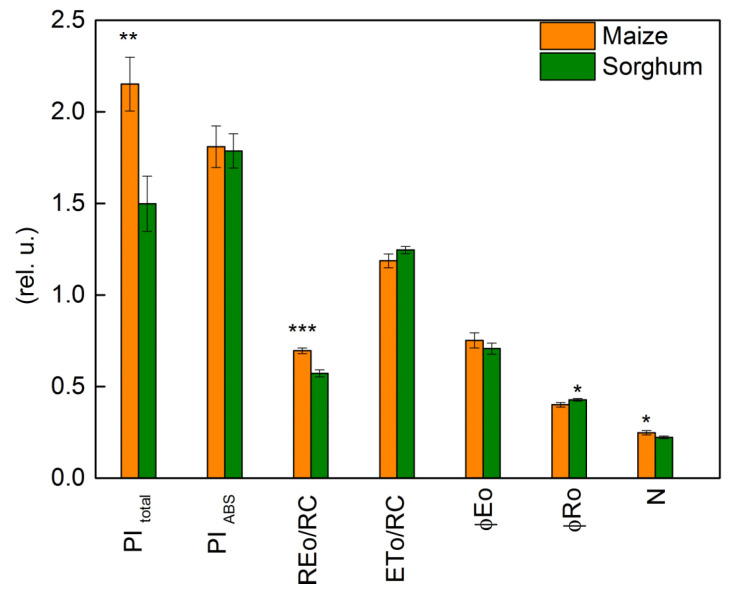
Selected JIP parameters in maize (*Zea mays* L. Mayflower) and in sorghum (*Sorghum bicolor* L. Foehn) under physiological conditions: performance index (potential) for energy conservation from exciton to the reduction in PSI end acceptors, PI _total_; performance index (potential) for energy conservation from exciton to the reduction in intersystem electron acceptors, PI _ABS_; electron flux reducing end electron acceptors at the PSI acceptor side per reaction center, REo/RC; electron transport flux (further than Q_A_) per reaction center, ETo/RC; quantum yield of electron transport (at t = 0), φEo; quantum yield of reduction in end electron acceptors at the PSI acceptor side, φRo; maximum turnovers of Q_A_ reduction until Fm was reached, N. Values (± SE) were calculated from 20 independent measurements. Asterisks indicate significant differences between maize and sorghum for the respective parameters (* *p* < 0.05, ** *p* < 0.01, *** *p* < 0.001).

**Figure 9:**
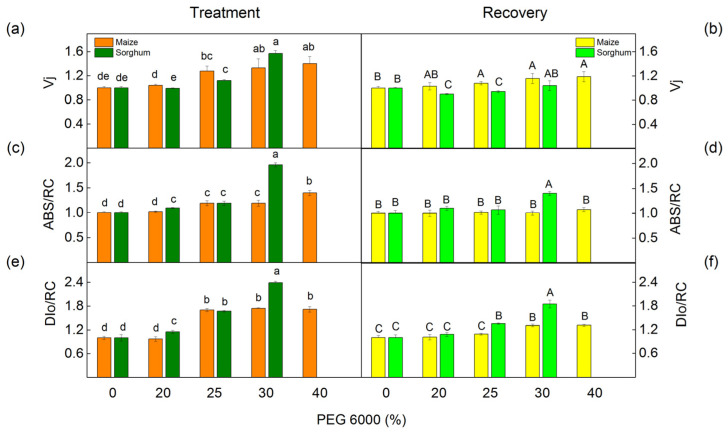
The selected JIP parameters in maize (*Zea mays* L. Mayflower) and in sorghum (*Sorghum bicolor* L. Foehn) after PEG treatment (**a**,**c**,**e**) and after the recovery period (**b**,**d**,**f**): relative variable fluorescence at the J step, Vj (**a**,**b**); absorption flux per reaction center, ABS/RC (**c**,**d**); dissipated energy flux per reaction center, DIo/RC (**e**,**f**). Mean values (±SE) are from 20 independent measurements. Different letters indicate significant differences for the respective parameters at *p* < 0.05 (lowercase for plants after the treatment and uppercase for plants after the recovery period).

Under physiological conditions, a comparison of two investigated species indicated insignificant variations in the JIP parameters: PI_ABS_, ETo/RC, φEo, ABS/RC, DIo/RC, and Vj (Figure 8 and Figure 9). At the same time, significant differences between control plants of sorghum and maize were registered in PI_total_, REo/RC, φRo, and N (Figure 8). In addition, the electron flux reducing end acceptors at the acceptor side of PSI (REo/RC), PI_total_, and N were bigger in maize than in sorghum (Figure 8).

**Figure 10:**
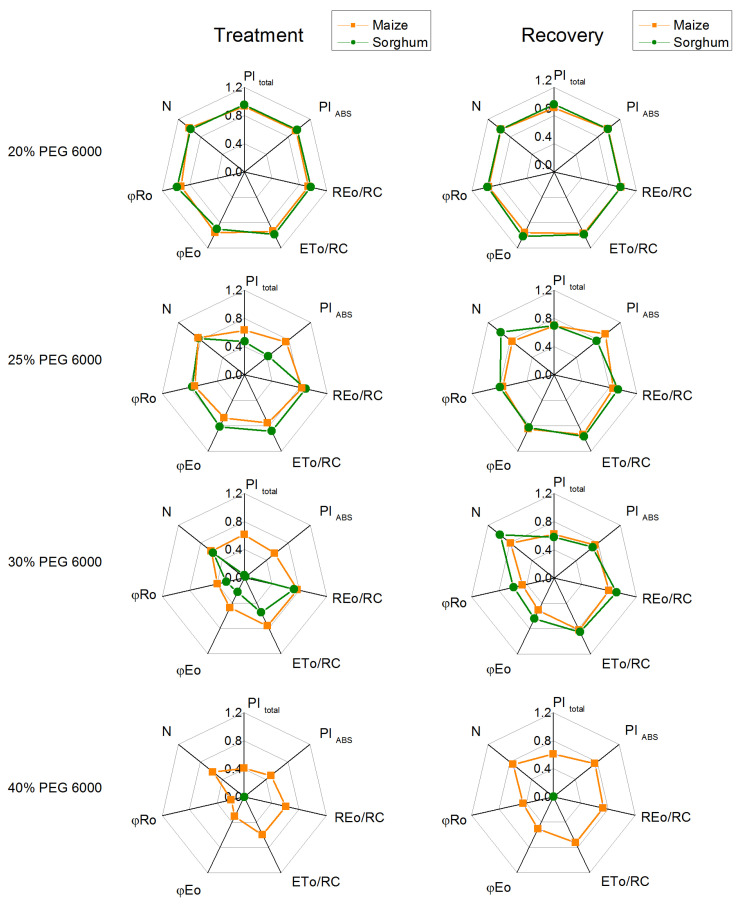
The selected OJIP parameters in maize (*Zea mays* L. Mayflower) and in sorghum (*Sorghum bicolor* L. Foehn) after PEG treatment and after the recovery period: performance index for energy conservation from exciton to the reduction in PSI end acceptors, PI_total_; performance index for energy conservation from exciton to the reduction in intersystem electron acceptors, PI_ABS_; electron flux reducing end electron acceptors at the PSI acceptor side per RC, REo/RC; electron transport flux (further than Q_A_) per reaction center, ETo/RC; quantum yield of electron transport (at t = 0), φEo; quantum yield of the reduction in end electron acceptors at the PSI acceptor side, φRo; maximum turnovers of Q_A_ reduction until Fm was reached, N. The parameters are normalized to the respective control. Mean values (±SE) are from 20 independent measurements.

The PEG treatment influenced the values of JIP parameters in maize and sorghum in different degrees in comparison with the values of the respective control plants (Figure 9 and Figure 10). Absorption flux per reaction center (ABS/RC), dissipated energy flux per reaction center (DIo/RC), and relative variable fluorescence at the J step (Vj) increased after treatment with 25% and higher PEG concentrations, as the effects were bigger in sorghum than in maize (Figure 9).

The data also revealed that the addition of 30% PEG to the nutrient solution influences the parameters PI_total_, PI_ABS_, REo/RC, ETo/RC, φEo, and φRo as the effects were more pronounced in sorghum than in maize, and after treatment with 25% PEG, significant differences were registered in the performance indices (PI_total_ and PI_ABS_) (Figure 10). At the same time, the treatment with 20% PEG led to negligible changes in PI_total_, PI_ABS_, REo/RC, ETo/RC, φEo, and φRo. The experimental results also showed that after the recovery period, the studied JIP parameters were restored to a different degree (Figure 9 and Figure 10).

### 2.7. P_700_ Photooxidation

**Figure 11:**
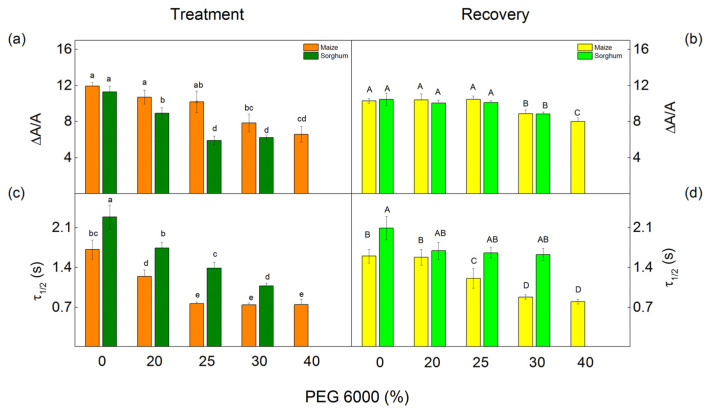
Effects of PEG 6000 and the recovery period on the relative changes in P_700_^+^ (∆A/A) (**a**,**b**) and half-time (*τ*_1/2_, s) (**c**,**d**) of the dark reduction of P_700_^+^ in maize (*Zea mays* L. Mayflower) and in sorghum (*Sorghum bicolor* L. Foehn). Means (±SE) were calculated from 8 independent measurements. Significant differences between treatments at *p* < 0.05 are shown in different letters (lowercase for the plants after the treatment and uppercase for the plants after the recovery period).

The redox properties of P_700_ were used to assess the effect of different PEG concentrations on PSI photochemistry. We investigated steady-state P_700_ photooxidation by the far-red light. It induced absorption changes of around 820 nm (ΔA/A) and the half-time (*τ*_1/2_) of the P_700_^+^ reduction in the dark. The photochemistry of PSI (measured as ΔA/A) was affected at different drought levels in the studied species (Figure 11). The parameter ΔA/A decreased in maize after treatment with 30% and 40% PEG, while an effect in sorghum was registered at all applied concentrations. After treatment with 30% PEG, the decrease in this parameter was more influenced in sorghum (from 21% to 45%) than in maize (from 10% to 34%). Drought also led to a decrease in the half-time (*τ*_1/2_) in both studied species; the decrease was from 27% to 56% in maize and from 24% to 53% in sorghum (Figure 11). After the recovery period, the values of the parameters ΔA/A were similar to the respective control for the plants treated with 20% and 25% PEG. In addition, after applying concentrations of 30% and 40% PEG to the nutrient solution, no full recovery of the photooxidation of P_700_ was observed (Figure 11b). The data also revealed an increase of *τ*_1/2_ in both studied species, as in sorghum, the values were similar to the control plants. This parameter in maize was smaller than in untreated plants excluding the plants treated with 20% PEG (Figure 11d).

## 3. Discussion

One of the most important environmental stress factors that has a negative impact on plant growth and development is drought [3,60,61]. The drought-induced changes in the plants depend on the water deficit level, durations, and plant species [62,63,64]. A typical symptom under drought that strongly changes the plant morphology is decreasing chlorophyll content [11,65,66]. Our experimental results revealed a decrease in the amount of pigments (Chl and Car) as the effect was more pronounced in sorghum than in maize (Table 1). This could be a result of enhanced pigment degradation [61,67] or the inhibition of the biosynthesis of chloroplast proteins, resulting in an inhibition of photosynthesis [7,68]. It has also been shown that the reduction of Chl *b* is bigger than that of Chl *a* [28]. The other reason for the drought-induced changes in Chl content could be an influence on the pigment biosynthesis [65,69]. The changes in chlorophylls were accompanied by an increase in the Chl *a*/*b* ratio (Table 2). A similar increase in this ratio was also observed in some plant tolerance species [28,70]. Previous studies revealed that the Chl *a*/*b* ratio correlates with the amount of LHCII and the degree of thylakoid membrane stacking [71,72,73]. It could be suggested that drought influences the organization of thylakoid membranes, i.e., decreases the degree of stacking. This assumption is also supported by studies that showed a modification in the thylakoid structure and granum under water deficit [15,64,74].

Moreover, drought led to a smaller decrease in Car content than in Chl content (Table 1). Data also revealed that the ratio of Car/Chl was affected in maize only at the highest concentration (40%), while in sorghum, insignificant influences were registered after applying all studied PEG concentrations (Table 2). It is known that one of the functions of Car is to act as an antioxidant and to protect membranes in plants from oxidative stress [75,76]. It could be suggested that a smaller influence on Car content under drought is one of the defense strategies in sorghum and in maize against the harmful effects of oxidative stress on the photosynthetic apparatus under water deficiency. The drought treatment led to an increase in anthocyanins in maize and in sorghum (Figure 2). The main roles of these pigments are in mediating responses to stress and light-screening properties [46]. It has been shown that the modulation of plant metabolism by anthocyanins leads to higher resistance under drought stress [46]. Anthocyanins also have an antioxidant capacity and scavenge the drought-induced ROS and also maintain osmotic balance [77,78]. The increase in anthocyanins under drought was reported in other plant species [46,79,80]. It could be supposed that the increased anthocyanin content after the PEG treatment is a defense strategy in studied species under drought.

Previous investigations have also shown that drought causes the accumulation of excessive ROS, which causes oxidative damage of the membranes [81,82]. The activity of antioxidant enzymes decreases the negative effects of drought stress [42]. Moreover, the secondary metabolites that participate in ROS detoxification and protein stabilization are also very important for plant drought resistance [43]. The present study revealed that PEG treatment leads to an increase in H_2_O_2_ content, lipid peroxidation (assessed by MDA content), and membrane injury index (MII) (Figure 2 and Table 3). The changes in these parameters depended on the drought level, and they were more strongly influenced in sorghum than in maize. The membrane injury can be used to assess the drought tolerance of the plants [83,84]. Smaller lipid peroxidation and electrolyte leakage in drought-tolerant genotypes of *Brassica napus* [64] and *Setaria italica* [85] have been shown. An important indicator of the influence of drought stress on plants is the RWC [86,87]. The data in this study revealed a strong decrease in RWC in studied species after treatment with higher PEG concentrations (25% and higher) (Figure 3).

The drought-induced changes in plants strongly influenced the primary reaction of the photosynthetic apparatus. The water deficiency decreased photochemical quenching (qP), which corresponds to the proportion of the open reaction centers, as in sorghum, the effect was stronger than in maize (Figure 4), which could be a result of the restriction of the electron flow between Q_A_^−^ and plastoquinone [51]. The analysis of the fluorescence induction curves showed that PEG treatment inhibited the electron transport flux from Q_A_ to Q_B_ per PSII (ET/RC) and electron flux reducing end electron acceptors at the PSI acceptor side per PSII reaction center (REo/RC) as well as a decrease in the relative size of the plastoquinone pool (N), which led to a decrease in the performance indexes PI_total_ and PI_ABS_ (Figure 10). Data also revealed an increase in the parameter Vj at higher PEG concentrations (Figure 9), which could be a result of the accumulation of the reduced Q_A_ and limitation of the electron transport beyond Q_A_ [88,89], which suggest the changes in the acceptor side of PSII [20,90]. These changes in the acceptor side of PSII are influenced by the drought-induced modification of the D1 and Q_B_ reducing complex, which influences the electron transfer between Q_A_ and Q_B_ [15,20]. The drought stress also decreased the ratio of the quantum yields of the photochemical to concurrent nonphotochemical processes in PSII (Fv/Fo), which inhibited the electron transport rate (ETR) (Figure 4). The decrease in the Fv/Fo ratio suggests structural changes in the thylakoid membrane [91]. Moreover, this ratio decreased stronger in sorghum than in maize (Figure 4); i.e., the drought-induced changes in thylakoid membranes in sorghum were bigger in comparison with maize. Some authors suggest that Fv/Fo corresponds to the efficiency of the OEC [92,93,94,95]; it can be concluded that water deficiency influences the donor side of PSII. The above results support the hypothesis of an effect of drought on the donor and acceptor sides of PSII [90,96].

Drought influences the stacking of the thylakoids and the organization of their protein complexes. Previous investigations have shown a reduction in the amount of PSII–LHCII supercomplexes, an increase in the LHCII monomers, a decrease in the PSII dimer, and changes in the organization of LHCII assemblies and their binding to the PSII core [97]. All these changes led to a decrease in the effective photochemical energy conversion quantum yield of PSII (Φ_PSII_) and an increase in the energy losses (the sum of Φ_NPQ_ and Φ_NO_) in both studied species (Figure 5 and Figure 6). Moreover, the changes in the energy losses in maize were a result of an increase in the regulated energy losses (Φ_NPQ_), while the sorghum drought led to a bigger increase in Φ_NO_ than in Φ_NPQ_. It has been suggested that the increased Φ_NO_ corresponds to an increased amount of singlet oxygen [98,99]. A comparison of the impact of PEG treatment on the studied species supposes a bigger amount of singlet oxygen in sorghum than in maize under drought. The main photoprotective process in the photosynthetic apparatus under abiotic stress is nonphotochemical quenching (NPQ) [100,101]. More information for the dissipation processes gives the components of NPQ, state transition (qT), and photoinhibitory quenching (qI) [102,103,104,105]. Data revealed that the increase in qT was bigger in maize than in sorghum (Figure 7). Having in mind that qT is important for the photoprotection of the photosynthetic apparatus [104,106], better protection of the photosynthetic apparatus could be suggested and could correspond to smaller drought-induced inhibition of the functions of the photosynthetic apparatus (Figure 4, Figure 5 and Figure 6). In support of this statement, there are also observed changes in qI (Figure 7) that can be used to assess PSII damage [100,104]. A stronger increase in this component (qI) in sorghum than in maize supposes bigger changes in the PSII complex of sorghum.

The impact of drought treatment on PSI (P_700_ photooxidation) was different in the studied plant species (Figure 11). The relative amount of P_700_^+^ (ΔA/A) in sorghum decreased after treatment with all studied PEG concentrations, while in maize, after applying 30% and 40% PEG. The changes in the parameter ΔA/A could be a result of drought-induced changes in the heterodimer of PSI [15,107]. At the same time, the water deficiency led to a decrease in the half-time *τ*_1/2_ in both studied species and all PEG concentrations (Figure 11). The observed changes in *τ*_1/2_ indicate an increase in the cyclic electron flow around PSI, which prevents the oxidative damage of the photosynthetic apparatus [108,109].

The data in this study revealed that after the recovery period (5 days), the negative effects of drought on the studied parameters decreased in both plant species. Experimental results revealed an increase in pigment content, a decrease in the markers of oxidative stress, and membrane injury, which correspond to decreased inhibition in the photochemical activity of PSII and PSI. In addition, the data showed better recovery in plants (sorghum and maize) treated with lower concentrations (20% and 25%) of PEG.

## 4. Materials and Methods

### 4.1. Plant Growth Conditions and Treatment

Plants of maize (*Zea mays* L. Mayflower) and sorghum (*Sorghum bicolor* L. Foehn) were used in this study. The seeds were obtained from Euralis Ltd. (Lescar, France). After germination, the plants were placed in boxes (15 plants in a box) with a half-strength Hoagland solution. The plants were grown in a photothermostat with controlled conditions, including 25 °C (daily)/23 °C (night) temperature, a light intensity of 150 µmol photons/m^2^ s, 12 h of light/dark photoperiod, and 65% humidity. After 10 days, different concentrations (20%, 25%, 30%, and 40%) of polyethylene glycol (PEG 6000) were added to the nutrient solution. The plants were treated with PEG for 3 days. The effects of different PEG concentrations on the studied plant species are shown in Figure S1.

To assess the ability of maize and sorghum to recover after drought, some of the plants were transferred to a nutrient solution without PEG for 5 days. The solutions were aerated constantly and were changed every 3 days. Two independent experiments (25–30 uniform plants for each treatment) were performed. The measurements and analysis were carried out on mature expanded leaves.

### 4.2. The Relative Water Content

The relative water content (RWC) was measured on the leaf segments, as described by Barrs and Weatherley [110]. The following parameters were determined: FW (fresh weight—immediately after cutting the leaves), TW (turgid weight—segments were put in distilled water to leaf water saturation), and DW (dry weight—after drying the leaves (at 80 °C for 24 h)). The following equation was used to calculate the RWC:

RWC (%) = (FW − DW)/(TW − DW) × 100

### 4.3. Photosynthetic Pigments

The amounts of chlorophyll *a* (Chl *a*), chlorophyll *b* (Chl *b*), and carotenoids (Car) were determined spectrophotometrically, as described by Stefanov et al. [105]. The pigments were extracted from leaves (30 mg) with 80% acetone in cold and dark conditions. The mixture was centrifuged at 4500× *g* for 10 min, and the absorption was measured at 663.2, 646.8, and 470 nm using a spectrophotometer (Specord 210 Plus, Edition 2010, Analytik Jena AG, Jena, Germany). For the calculation of the amounts of the pigments, the equations of Lichtenthaler were used [111].

### 4.4. Anthocyanin Content

The anthocyanins were determined, as described by Murray and Hackett [112]. The extraction was made with a medium containing C_2_H_5_OH:HCl:H_2_O at a ratio of 79:1:20. The prepared leaf suspension was centrifuged at 10,000× *g* for 15 min. The absorbance was measured at 535 and 653 nm on a spectrophotometer (Specord 210 Plus, Edition 2010, Analytik Jena AG, Jena, Germany). The following equation was used for the determination of the anthocyanin content: A_535_ − 0.24 × A_653_.

### 4.5. Determination of Oxidative Stress Markers and Membrane Injury Index

The amounts of hydrogen peroxide (H_2_O_2_) and malondialdehyde (MDA) were determined, as described by Yotsova et al. [113]. The H_2_O_2_ content was estimated after its colorimetric reaction with KI at 390 nm absorbance (Specord 210 Plus, edition 2010, Analytik Jena AG, Jena, Germany), using the molar extinction coefficient 0.28 µM^−1^ cm^−1^. The MDA level was determined using thiobarbituric acid and a molar extinction coefficient of 0.155 µM^−1^ cm^−1^. The amounts of H_2_O_2_ and MDA were expressed as nmol per g DW.

The membrane injury index (MII) was determined, as described previously in [114]. Mature leaves were cut into small leaf fragments (averaged 4 cm^2^ leaf area) and incubated in a tube with distilled water for 24 h at room temperature in the dark and determined the electrical conductivity (T1 and C1). After that, the samples were boiled (30 min) and cooled (25 °C) to determine the electrical conductivity (T2 and C2). For measurements of the electrical conductivity, a conductometer (Hydromat LM302, Witten, Germany) was used. The following equation was used to calculate the membrane injury index:

MII (%) = [1 − (1 − T1/T2) × (1 − C1/C2)] ×100

where T1 and T2 are the first and second (after boiling) conductivity of the solutions with the treated plant leaf samples, and C1 and C2 are the values from the leaves of the controls (untreated plants) [114].

### 4.6. Chlorophyll Fluorescence Measurements

The pulse-amplitude-modulated (PAM) chlorophyll fluorescence was measured on leaves using a PAM fluorimeter (H. Walz, Effeltrich, Germany, model PAM 101-103). The Fo level was measured at an instrument frequency of 1.6 kHz, and the measuring beam was set at 0.02 μmol photons/m^2^ s. The maximal fluorescence levels Fm and Fm′ were measured using a saturating pulse illumination of 3000 μmol photons/m^2^ s, which was provided by Schott lamp KL 1500 (Schott Glaswerke, Mainz, Germany). The actinic light (150 μmol photons/m^2^ s) was provided by a second Schott lamp KL 1500 [58]. The following parameters were estimated: the ratio of quantum yields of the photochemical and concurrent nonphotochemical processes in PSII (Fv/Fo = (Fm − Fo)/Fo; the photochemical quenching, qP = (Fm′ − Fs)/Fv′; the effective quantum yield of PSII photochemistry, Φ_PSII_ = (Fm′ − Fs)/Fm′; the relative PSII electron transport rate, ETR = Φ_PSII_ × PFD × 0.5 [115]; the nonregulated (Φ_NO_ = Fs/Fm) and regulated (Φ_NPQ_ = Fs/Fm′ − Fs/Fm) energy loss in PSII; the components of the nonphotochemical quenching: the state transition quenching, qT; and the photoinhibitory quenching, qI [58].

The chlorophyll fluorescence induction curves were measured using a Handy PEA+ (Hansatech, Norfolk, UK). The measurements were performed by leaf clips after 20 min of dark adaption. The intensity of the light pulse was 3000 μmol photons/m^2^ s. The duration of the measurement lasted 3 s. These measurements were repeated 20 times per variant. All studied variants showed a multiphase increase in chlorophyll fluorescence. The measured data were used to calculate the selected JIP test parameters [116,117,118]: ABS/RC—specific absorption flux per reaction center (RC), i.e., effective antenna size of an active RC; ETo/RC—electron transport flux per RC further than Q_A_; REo/RC—electron flux per active RC reducing the end electron acceptors on the acceptor side of PSI (at t = 0); DIo/RC—dissipated energy flux per RC (at t = 0); φEo—quantum yield of electron transport (at t = 0); φRo—quantum yield of reduction in end electron acceptors at the PSI acceptor side; Vj—relative variable fluorescence at the J step; N—maximum turnovers of Q_A_ reduction until Fm was reached; PI_ABS_—performance index (potential) for energy conservation from exciton to the reduction in intersystem electron acceptors; PI_total_—performance index (potential) for energy conservation from exciton to the reduction of PSI end acceptors.

### 4.7. P_700_ Photooxidation

The redox state of P_700_ was determined by a PAM 101/103 fluorometer (Walz, Effeltrich, Germany) connected to an emitter–detector (ED-800T), as described by Dobrikova et al. [119]. Detached leaves (after dark adaptation) were irradiated with far-red (FR) light for 20 s emitted by a photodiode (102-FR, Walz, Effeltrich, Germany) to examine the absorbance changes at 830 nm (∆A/A) and the half-time of dark reduction in P_700_^+^ (*τ*_1/2_) [58].

### 4.8. Statistics

Data were shown as mean values (±SE). The means were calculated from at least two independent experiments with four replicates of each variant. Statistically significant differences between variants of the studied parameters were identified by two-way analysis of variance (ANOVA), followed by Tukey’s post hoc test for each parameter. Values of *p* < 0.05 were considered significantly different.

## 5. Conclusions

In conclusion, the present study revealed that drought treatment decreased the open reaction centers of PSII (qP), the effective quantum yield of the photochemical energy conversion in PSII (Φ_PSII_), the rate of electron transport (ETR), the efficiency of the OEC, and the performance indices PI _total_ and PI _ABS_, and these processes were stronger influenced in sorghum than in maize., which suggests the different drought tolerances of these crop species. Water deficiency influenced the photochemistry of PSI in both studied species, but the effect was observable at smaller PEG concentrations in sorghum than in maize. The observed changes are probably the result of a bigger disruption of membrane integrity in sorghum in comparison with maize. The data also revealed better postdrought recovery in plants of both species treated with low concentrations of PEG (20% and 25%). The experimental results in this study clearly showed the high sensitivity of the primary photosynthetic processes under different drought levels; therefore, the changes in these processes could be used for assessing the sensitivity and degree of damage of the plants under drought. The increase in the regulated energy losses (Φ_NPQ_), the induction of the state transition (qT), and the cyclic electron flow around PSI provide better protection of the photosynthetic apparatus; therefore, these processes could be used as indicators of the drought tolerance of the plants.

## Figures and Tables

**Table 1:** The amounts of leaf total chlorophyll (Chl) and carotenoid (Car) content in maize (*Zea mays* L. Mayflower) and in sorghum (*Sorghum bicolor* L. Foehn) after PEG treatment and after the recovery period of the drought-treated plants. Mean values (±SE) were calculated from 8 independent measurements. Different letters indicate significant differences among treatments at *p* < 0.05 (lowercase for the plants after the treatment and uppercase for the plants after the recovery period). *—lethal PEG concentration.

PEG 6000 (%)	Chl (mg/g DW)		Car (mg/g DW)	
	Treatment	Recovery	Treatment	Recovery
*Zea mays* L.				
0	25.44 ± 0.28 ^c^	25.44 ± 0.28 ^C^	4.78 ± 0.51 ^b^	4.78 ± 0.51 ^C^
20	22.95 ± 0.27 ^d^	22.20 ± 0.25 ^D^	4.62 ± 0.69 ^b^	4.80 ± 0.36 ^B^
25	12.43 ± 0.36 ^f^	17.56 ± 0.44 ^E^	2.82 ± 0.05 ^c^	3.87 ± 0.32 ^C^
30	12.38 ± 0.33 ^f^	15.20 ± 0.37 ^F^	2.89 ± 0.09 ^c^	3.89 ± 0.15 ^C^
40	7.90 ± 0.13 ^g^	9.70 ± 0.25 ^G^	2.03 ± 0.06 ^d^	3.08 ± 0.14 ^D^
*Sorghum bicolor* L.				
0	33.59 ± 0.14 ^a^	33.59 ± 0.14 ^A^	6.13 ± 0.30 ^a^	6.13 ± 0.30 ^A^
20	27.71 ± 0.62 ^b^	30.65 ± 1.44 ^AB^	6.87 ± 0.15 ^a^	6.09 ± 0.19 ^A^
25	17.51 ± 0.30 ^e^	27.69 ± 0.80 ^B^	4.07 ± 0.13 ^b^	5.51 ± 0.10 ^AB^
30	11.58 ± 0.28 ^f^	21.89 ± 1.69 ^D^	3.86 ± 0.08 ^b^	4.73 ± 0.40 ^BC^
40	*	*	*	*

**Table 2:** The pigment ratios Chl *a*/*b* and Car/Chl in maize (*Zea mays* L. Mayflower) and in sorghum (*Sorghum bicolor* L. Foehn) after PEG treatment and after the recovery period of the drought-treated plants. Mean values (±SE) were calculated from 8 independent measurements. Different letters indicate significant differences among treatments at *p* < 0.05 (lowercase for the plants after the treatment and uppercase for the plants after the recovery period). *—lethal PEG concentration.

PEG 6000 (%)	Chl *a*/*b*		Car/Chl	
	Treatment	Recovery	Treatment	Recovery
*Zea mays* L.				
0	3.40 ± 0.14 ^c^	3.40 ± 0.14 ^B^	0.20 ± 0.02 ^bc^	0.20 ± 0.02 ^BC^
20	3.88 ± 0.03 ^b^	3.78 ± 0.09 ^AB^	0.20 ± 0.02 ^bc^	0.22 ± 0.01 ^B^
25	3.72 ± 0.09 ^bc^	3.83 ± 0.06 ^AB^	0.23 ± 0.03 ^bc^	0.22 ± 0.03 ^BC^
30	3.90 ± 0.03 ^b^	3.76 ± 0.36 ^AB^	0.23 ± 0.02 ^bc^	0.26 ± 0.03 ^AB^
40	4.15 ± 0.24 ^ab^	4.06 ± 0.20 ^A^	0.26 ± 0.03 ^ab^	0.32 ± 0.03 ^A^
*Sorghum bicolor* L.				
0	3.81 ± 0.16 ^bc^	3.81 ± 0.16 ^AB^	0.18 ± 0.01 ^c^	0.18 ± 0.01 ^C^
20	4.18 ± 0.21 ^ab^	4.07 ± 0.23 ^A^	0.25 ± 0.01 ^b^	0.20 ± 0.01 ^BC^
25	4.24 ± 0.06 ^a^	4.30 ± 0.31 ^A^	0.23 ± 0.03 ^bc^	0.20 ± 0.01 ^BC^
30	4.16 ± 0.12 ^ab^	4.26 ± 0.10 ^A^	0.33 ± 0.01 ^a^	0.22 ± 0.02 ^BC^
40	*	*	*	*

**Table 3:** Membrane injury index in maize (*Zea mays* L. Mayflower) and in sorghum (*Sorghum bicolor* L. Foehn) after treatment with different PEG concentrations and after the recovery period. Mean values (±SE) were calculated from 8 independent measurements. Different letters indicate significant differences among treatments at *p* < 0.05 (lowercase for the plants after the treatment and uppercase for the plants after the recovery period). *—lethal PEG concentration.

PEG 6000 (%)	Membrane Injury Index (%)	
	Treatment	Recovery
*Zea mays* L.		
20	20.19 ± 1.26 ^e^	10.99 ± 1.09 ^E^
25	33.48 ± 1.51 ^d^	21.80 ± 1.49 ^D^
30	49.91 ± 3.96 ^c^	27.61 ± 1.81 ^C^
40	56.63 ± 3.82 ^b^	34.66 ± 2.27 ^A^
*Sorghum bicolor* L.		
20	36.43 ± 1.79 ^d^	30.35 ± 1.99 ^B^
25	70.91 ± 2.55 ^a^	34.12 ± 1.79 ^A^
30	70.99 ± 4.00 ^a^	34.13 ± 2.18 ^A^
40	*	*

## Data Availability

Not applicable.

## Supplementary Materials

The following supporting information can be downloaded at: https://www.mdpi.com/article/10.3390/plants12091863/s1, Figure S1: Effects of different concentrations of PEG 6000 on maize (*Zea mays* L. Mayflower) and sorghum (*Sorghum bicolor* L. Foehn). The time of the treatment was 3 days.
