# Supplementary material for: Sensitivity of the Photosynthetic Apparatus in Maize and Sorghum under Different Drought Levels
Source: Plants (Basel). 2023 Apr 30;12(9):1863. doi: 10.3390/plants12091863 (PMC10180982; doi:10.3390/plants12091863)
Supplement: Supplementary file 1 [file plants-12-01863-s001.zip › plants-2314756-supplementary.pdf]

Sensitivity of the Photosynthetic Apparatus in Maize and Sorghum under Different Drought Levels

Martin Stefanov, Georgi Rashkov, Preslava Borisova and Emilia Apostolova

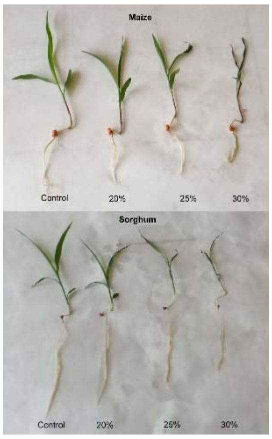

Figure S1. Effects of different concentrations of PEG 6000 on maize (*Zea mays* L. Mayflower) and sorghum (*Sorghum bicolor* L. Foehn). The time of the treatment was 3 days.

Visualization of maize seedlings (roots, stems, leaves) (A) and sorghum seedlings (roots, stems, leaves) (B) after three days of growth in nutrient solutions containing different concentrations of PEG 6000
